# To Medical Freemasons

**Published:** 1912-09-21

**Authors:** William Wilson

**Affiliations:** Secretary, St. Luke's Medical Lodge of Instruction. 184 Goldhawk Road, W.


*To the Editor of* The Hospital.

### To Medical Freemasons.

*To the Editor of* The Hospital.

Sir,—May I through the medium of your widely read columns appeal to medical men, and medical Freemasons in particular, for votes for the Masonic charities? At the forthcoming elections there are several medical candidates. Votes for any of the Masonic institutions are equally acceptable, as exchanges can be effected, and will be gratefully received and acknowledged by me.—I am, Sir, yours faithfully, William Wilson,

Secretary, St. Luke's Medical Lodge of Instruction.

184 Goldhawk Road, W., September 17.

